# Gut Microbiota Modulation by Carboxymethyl Cellulose and Carrageenan: Current Evidence and Health Implications

**DOI:** 10.3390/foods15081437

**Published:** 2026-04-20

**Authors:** Ana Fernandes, Débora A. Campos, Ezequiel R. Coscueta, Maria Manuela Pintado

**Affiliations:** Universidade Católica Portuguesa, CBQF—Centro de Biotecnologia e Química Fina—Laboratório Associado, Escola Superior de Biotecnologia, Rua Diogo Botelho 1327, 4169-005 Porto, Portugal; aclpfernandes@ucp.pt (A.F.); mpintado@ucp.pt (M.M.P.)

**Keywords:** carrageenan, carboxymethyl cellulose, gut microbiota, inflammation

## Abstract

The gut microbiota plays a central role in digestion, metabolism, immune regulation, and inflammatory processes, and is highly responsive to dietary factors, including food additives. With the increasing consumption of ultra-processed foods, growing attention has been directed toward the long-term effects of commonly used additives on gut health. This review examines the interactions between food additives and the gut microbiota, with a specific focus on the emulsifiers carboxymethyl cellulose (CMC) and carrageenan (CGN), which are widely used in processed foods. Evidence from in vitro, animal, and limited human studies indicates that both CMC and CGN can alter gut microbiota composition, disrupt intestinal barrier integrity, and promote pro-inflammatory responses, although their mechanisms of action differ. CGN has been more consistently associated with direct activation of inflammatory signalling pathways and epithelial stress, whereas CMC primarily induces microbiota-mediated effects, including altered microbial spatial organisation and mucus barrier disruption, leading to low-grade inflammation. The magnitude of these effects appears to depend on dosage, duration of exposure, and the experimental model employed. Overall, the findings summarised in this review suggest that chronic exposure to CMC and CGN may contribute to gut dysbiosis and increased inflammatory susceptibility, particularly within dietary patterns rich in ultra-processed foods. These observations highlight the need for harmonised research methodologies, more human-relevant long-term studies, and reconsideration of current food safety assessment frameworks to better account for microbiota-related outcomes.

## 1. Introduction

The human gut microbiota is a complex ecosystem of microorganisms colonizing the digestive tract and plays a crucial role in digestion, metabolism, immune function, and disease prevention [1]. Firmicutes and Bacteroidota dominate the gut microbiome, with the genera *Clostridium* and *Bacteroides* as key representatives [2]. Maintaining a balanced microbial composition is essential for health, as dysbiosis—an imbalance in gut microbiota—has been linked to various conditions, including obesity, colorectal cancer, and type II diabetes, among other chronic diseases [3,4]. Multiple external factors influence the gut microbiota, including diet, lifestyle, and medications [5]. One growing concern is the effect of food additives—substances that enhance visual aspect, flavour, taste, texture, and shelf life—on microbial composition [5]. While regulatory bodies consider many additives safe, emerging research suggests that some may disrupt gut homeostasis, potentially contributing to inflammation and disease [4,5]. Despite increasing interest in the gut microbiome, the impact of food additives remains understudied compared to macronutrients and other dietary components.

This review examines current evidence on the interactions between food additives and the gut microbiota, highlighting their potential role in disease progression. By summarising existing literature and identifying research gaps, this work aims to provide insights into the broader implications of food additives on human health.

## 2. The Human Gut Microbiota Composition and Interaction

**Figure 1:**
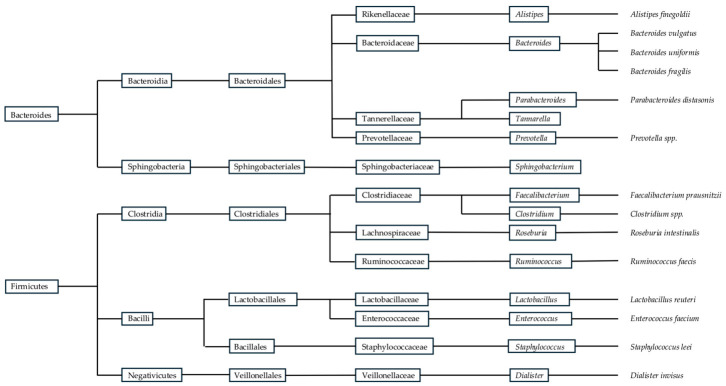
Taxonomic tree diagram of the most prominent bacteria within the Bacteroidota and Firmicutes phyla on gut microbiota, adapted from [1,5].

The gut microbiota is a thriving ecosystem of microorganisms that performs intricate processes that profoundly influence our health and well-being, changing with the host. Therefore, the gut microbiota evolves into a diverse community of microorganisms. According to numerous studies, the human gut microbiota contains more than 1000 microbial species [1,5]. The composition of an individual’s gut microbiota can vary significantly from person to person, influenced by factors such as genetics, diet, age, and environment; it also differs along the gastrointestinal tract, leading to the assembly of specific communities [6]. The main phyla in the gut microbiota are Bacteroidota, Firmicutes, Fusobacteria, Proteobacteria, Cyanobacteria, Verrucomicrobia, and Actinobacteria [6]. The most common species of these phyla are presented in the figure below (Figure 1).

**Figure 2:**
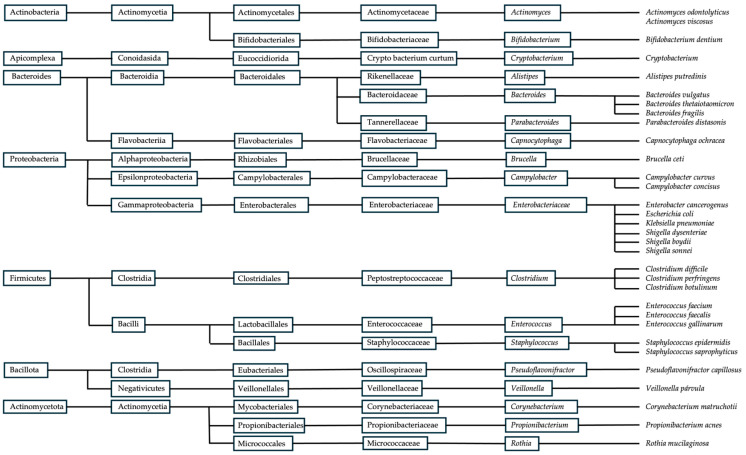
Taxonomic tree of pathogenic microorganisms present in the healthy gut microbiota, adapted from [7].

The Human Microbiome Project Consortium identified pathogenic species in healthy individuals using shotgun metagenomic data and unique marker sequences. This analysis, which encompasses the largest cohort and most diverse collection of clinically relevant body habitats to date, offers significant insights into the complexity of human-associated microbial communities [7]. Although several pathogenic species (Figure 2) may also be present in healthy individuals, their presence alone does not necessarily indicate a pathological condition. Instead, evaluating shifts in their relative abundance is essential when interpreting gut microbiota alterations associated with serious diseases. Both increases and decreases in these taxa may reflect significant changes in microbial community structure and function. Therefore, examining their relative proportions, ecological interactions, and collective influence on the microbial ecosystem is crucial for accurately characterizing gut microbiome dynamics. The human gut microbiota is largely dominated by the phyla Firmicutes and Bacteroidota. The phylum Firmicutes includes more than 200 genera, such as *Lactobacillus*, *Bacillus*, *Clostridium*, *Enterococcus*, and *Ruminococcus*, with many members belonging to the class Clostridia, which constitutes a substantial proportion of this phylum. In contrast, the phylum Bacteroidota is mainly represented by the dominant genera *Prevotella* and *Bacteroides* [7]. Furthermore, according to Mariat et al. (2009) [7], the dominant genera characterising the adult faecal microbiota include *Clostridium* cluster IV (e.g., *Faecalibacterium*), *Clostridium* cluster XIVa (e.g., *Roseburia*, *Eubacterium*), *Bacteroides*, and *Bifidobacterium*. In contrast, genera such as *Lactobacillus*, *Escherichia*/*Shigella* (representing *Enterobacteriaceae*), *Desulfovibrio*, *Sporomusa*, *Atopobium*, and other less abundant members of *Clostridium* clusters XI, XIVb, and XVIII constitute secondary components of the microbiota [7].

The abundance of Firmicutes in the gut microbiota of healthy individuals ranges from 11% to 95%, while Bacteroidota varies from 0.6% to 86.6%. Thus, the relative abundance of these phyla is highly variable even among subjects within the same population [2]. This variability is expected, as the gut microbiota is influenced by factors such as diet, physical activity, food additives, contaminants, and antibiotic use [8]. Bacteroidota play a crucial role in protecting against pathogens and supplying nutrients to other microbial residents, thereby contributing to immune system stability. They are primarily responsible for producing short-chain fatty acids (SCFAs), mainly acetate and propionate, which serve as potent anti-inflammatory mediators by reducing the release of pro-inflammatory cytokines from neutrophils and macrophages [8,9].

Moreover, Bacteroidota can thrive over a wide pH range and modify the nutritional landscape by synthesising compounds such as fucosylated glycoproteins or by releasing fucose and sialic acid residues from glycoproteins, which can then be utilised by other microorganisms, including pathogens [9,10]. The Firmicutes phylum tends to be stable under neutral or slightly acidic conditions, adapting to the specific gastrointestinal tract regions in which it resides. Firmicutes are fundamental to various metabolic pathways in the gut, encompassing the metabolism of dietary nutrients, bile acids, and host-derived compounds. Their ability to metabolise a wide range of substrates significantly influences nutrient absorption, energy balance, and the production of bioactive molecules [11]. It has also been observed that the balance between these two phyla has been associated with the maintenance of intestinal homeostasis, with imbalances being linked to several pathologies, including obesity, colorectal cancer, and type II diabetes [12].

**Figure 3:**
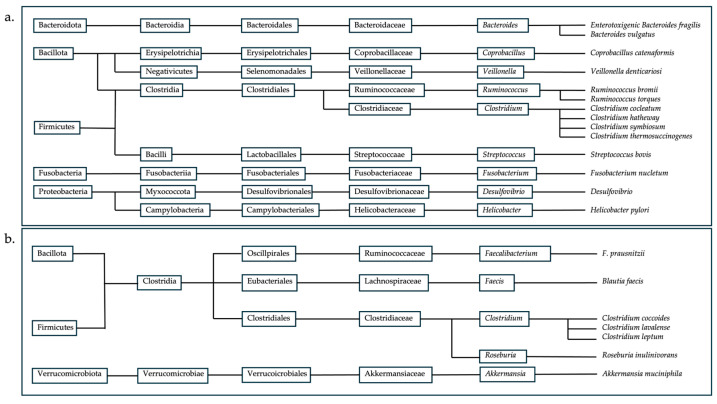
Taxonomic tree of microorganisms whose (**a**) increase leads to pathogeny and whose (**b**) decrease leads to pathogeny in the human gut; adapted from [8].

Such dysbiosis is typically characterized by shifts in the abundance of specific microbial taxa. For example, increases in genera such as *Enterococcus*, *Fusobacterium*, *Parvimonas*, *Gemella*, and *Leptotrichia*, as well as members of the family *Enterobacteriaceae*, have frequently been reported in disease conditions. In contrast, beneficial genera such as *Lachnospira*, *Clostridium*, *Bifidobacterium*, *Faecalibacterium*, *Blautia* and *Roseburia* are often reduced. Alterations in the abundance of these taxa, either through overgrowth or depletion, can disrupt microbial community stability and contribute to pathologies associated with gut dysbiosis (Figure 3).

## 3. Gut Microbiota and Its Impacts on Human Health

According to the literature, the gut microbiota contributes significantly to human physiology through its metabolic activities [6,13]. These microorganisms decompose complex carbohydrates and proteins by producing a variety of enzymes essential for nutrient metabolism [6]. In addition to its metabolic functions, the gut microbiota plays a pivotal role in modulating the human immune system. It regulates nutrient absorption, host metabolism, and influences gene expression and cellular physiology, thereby modulating immune responses [14].

**Figure 4:**
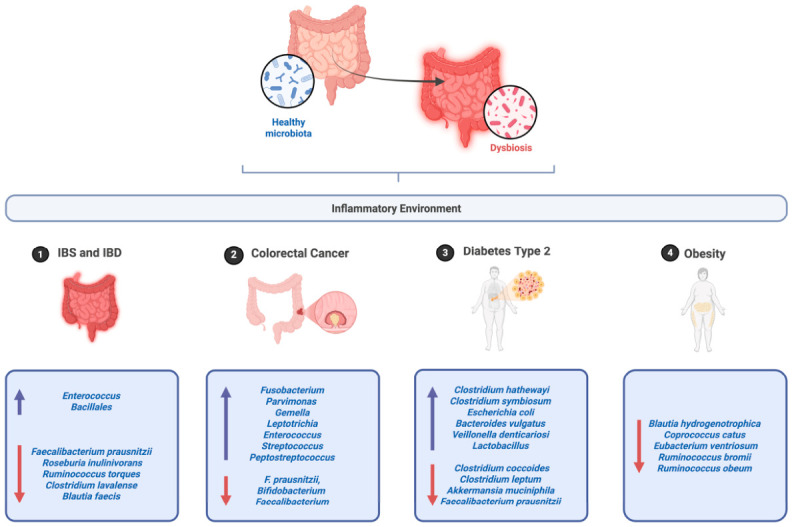
Schematic representation of the shift from healthy microbiota to dysbiosis, contributing to chronic inflammation and the development of gastrointestinal and metabolic diseases (upwards arrows representing increase, and downwards arrows representing decrease). Created in BioRender. Coscueta, E. (2026) https://BioRender.com/8c35gqq (accessed on 11 March 2026).

This intricate connection supports innate and adaptive immune functions and is closely linked to overall human health. Moreover, its involvement in the digestion and absorption of nutrients via fermentation and colonic decomposition has been well documented [15]. For instance, the relative abundance of *Ruminococcus 2*, *Faecalibacterium*, and *Akkermansia* is associated with increased neutrophil rates, and the total abundance of *Faecalibacterium* correlates with neutrophil dynamics, underscoring the critical role of the gut microbiota in immune function [15]. Furthermore, dysbiosis of the gut microbiota is implicated in various diseases, including inflammatory bowel disease (IBD), irritable bowel syndrome (IBS), diabetes, obesity, cancer, cardiovascular problems, and central nervous system disorders (Figure 4). Therefore, understanding the mechanisms by which the microbiome contributes to these conditions is paramount.

- **Inflammatory Bowel Disease and Irritable Bowel Syndrome**

IBD and IBS are chronic gastrointestinal disorders that affect the gastrointestinal tract (GIT). In the case of IBD, the main forms include Crohn’s disease and ulcerative colitis. Individuals with IBD often exhibit dysbiosis, characterized by alterations in the abundance and diversity of specific microbial taxa in the gut [16]. IBS is a common GIT disorder that affects the large intestine (colon), resulting in abnormal intestinal motility and low-grade inflammation. Several studies have linked these syndromes to gut microbiota dysbiosis, which may contribute to the intestinal inflammation observed, particularly in IBD and, to a lesser extent, in IBS [16,17].

It is essential to understand which alterations in the microbiota are associated with the development of these conditions. In both diseases, the gut microbiota is primarily composed of the phyla Firmicutes and Bacteroidota. In IBS specifically, an increased abundance of *Enterococcus* and members of the order Bacillales has been observed, along with an overall increase in Bacteroidota and a relative reduction in Firmicutes. This reduction notably includes *Faecalibacterium prausnitzii*, a butyrate-producing bacterium known for its anti-inflammatory properties. Consequently, this decrease may lead to reduced concentrations of SCFA [16,17]. Nevertheless, in IBD patients, the impact of dysbiosis often depends on the location within the GIT. Microbial communities typically show higher phylogenetic diversity in the large intestine compared with the small intestine. In the small intestine, there is a higher abundance of *Lactobacillus*, while members of the family Lachnospiraceae are present at lower levels compared with the colon.

Throughout the GIT, patients with IBD exhibit a decrease in species such as *Blautia faecis*, *Roseburia inulinivorans*, *Ruminococcus torques*, and *Clostridium lavalense*. Conversely, an increased abundance of *Desulfovibrio*, a genus of sulfate-reducing bacteria, has been observed. These bacteria can damage intestinal epithelial cells and promote mucosal inflammation. Additionally, a subtle increase in Proteobacteria—particularly *Escherichia coli* strains with adherent-invasive characteristics (AIEC)—has been associated with enhanced intestinal mucosal pro-inflammatory responses [16,18,19,20]. A significant decrease in the abundance of *Faecalibacterium* and *Bifidobacterium* has been observed exclusively in IBS, with the gut microbiota predominantly dominated by Enterobacteriaceae. This shift is associated with a higher prevalence of pro-inflammatory phylotypes, including *Clostridium cocleatum* (88%), *Clostridium thermosuccinogenes* (85%), *Coprobacillus catenaformis* (91%), *Ruminococcus bromii*-like (91%), and *Ruminococcus torques* (93%) [17,21].

**ii.**  **Colorectal Cancer**

Colorectal cancer (CRC) occurs when cells grow in the large intestine, making it one of the most common cancers worldwide [22,23]. Growing evidence suggests that gut dysbiosis is associated with CRC progression, as multiple studies have identified specific pathogenic bacterial taxa with carcinogenic potential [24]. According to tissue and stool assays, it is possible to identify that genera, such as *Fusobacterium*, *Parvimonas*, *Gemella*, *Leptotrichia*, *Enterococcus*, *Escherichia*/*Shigella*, *Klebsiella*, *Streptococcus*, and *Peptostreptococcus,* are enriched; and anti-inflammatory *F. prausnitzii*, *Bifidobacterium*, *Faecalibacterium*, and *Blautia* are in lower abundance in CRC patients [20,24]. Furthermore, enterotoxigenic *Bacteroides fragilis* (ETBF), which encodes *B. fragilis* metalloprotease toxin (BFT) to induce diarrhoea in most reports, and *Fusobacterium nucletum*, which has proven to be invasive, are highly expressed in CRC tissue when compared with healthy tissue [18,24].

On a separate issue, CRC patients also exhibit significantly lower SCFA abundance and SCFA-producing bacteria, which may be driven by a reduction in Firmicutes and an increase in Bacteroidota, leading to the development and progression of CRC [25].

**iii.**  **Diabetes Type 2**

Research suggests that the composition and function of the gut microbiota may contribute to the development and progression of Type 2 diabetes (T2DM). While healthy individuals have a high abundance of butyrate-producing bacteria, patients with T2DM exhibit reductions primarily in *Clostridium coccoides*, *Clostridium leptum*, *Akkermansia muciniphila*, and *Faecalibacterium prausnitzii*. Conversely, they show increased levels of several pathogenic bacteria, including *Clostridium hathewayi*, *Clostridium symbiosum*, *Escherichia coli*, *Bacteroides vulgatus*, *Veillonella denticariosi*, and *Lactobacillus*. Furthermore, the genera *Bifidobacterium*, *Bacteroides*, *Faecalibacterium*, *Akkermansia*, and *Roseburia* are negatively associated with T2DM, whereas the genera *Ruminococcus*, *Fusobacterium*, and *Blautia* are positively associated with the disease [26,27,28]. Moreover, when analysing individuals by glucose tolerance status, T2DM patients have a higher abundance of *Lactobacillus* species and a lower abundance of *Clostridium* species than individuals with normal glucose tolerance. While *Lactobacillus* has been positively associated with fasting glucose, *Clostridium* species have been negatively correlated with it, suggesting that these same bacterial taxa are associated with the development of T2DM [27,28].

**iv.**  **Obesity**

According to several studies, obesity can be influenced by the imbalance of the Firmicutes/Bacteroidota ratio at the phylum level, showing a low amount of Firmicutes, such as *Blautia hydrogenotrophica*, *Coprococcus catus*, *Eubacterium ventriosum*, *Ruminococcus bromii*, and *Ruminococcus obeum*, with a prevalence of *Prevotella* in obese groups [17,29]. Moreover, since *Methanobacteriales*, *Lactobacillus*, *Bifidobacteria* genera, and Christensenellaceae and Akkermansia families are used as probiotics to help this condition, it is essential to analyse their reduction, as it can be associated with obesity [19].

## 4. Food Additives Related to Gut Health Issues

Nowadays, our products are often far from their natural state, mainly presented in altered forms. This is due to the pressure to ensure specific quality, convenience and shelf life parameters that must be maintained to increase food products’ time-to-market. Food additives are crucial players, as they are intentionally incorporated into products to achieve specific goals, such as preserving food, enhancing colour and flavour, improving texture, or extending shelf life. According to the Codex Alimentarius, an additive is defined as any substance that would not be usually used as an ingredient but is applied in food for manufacture, processing, preparation, treatment, packing, packaging, transport, or holding purposes, which may or may not maintain or improve its nutritional qualities [30]. Since they have become normalised in human consumption, the potential health issues they may cause have been studied. Some of them were blocked from use in food products due to their hazardous effects, which have the potential to cause gut dysbiosis. Considering this, research on this topic has been conducted to understand the impact of additives (still circulating) on our health [5].

Many food additives could have effects like those discussed, but the additives listed below (Table 1) are currently under research by the European Food Safety Authority (EFSA) [5]. Among these additives, Carrageenan (CGN; E407) and Carboxymethyl cellulose (CMC; E466) have been shown to pose potential risks to the gut microbiota, highlighting the need to deepen our understanding of their toxicological properties and their capacity to disrupt microbial homeostasis, potentially leading to dysbiosis-associated pathological conditions.

## 5. Gut Modulation by CGN and CMC: Insights and Implications

The literature reviewed highlights the distinct effects of CGN and CMC on gut health. Studies indicate that both additives can alter gut microbiota composition, disrupt the intestinal barrier, and promote inflammatory responses.

It is important to note that the available literature on CGN and CMC is characterised by substantial methodological heterogeneity, including differences in experimental models (in vitro, animal, and human), sample size, exposure duration, additive form and molecular characteristics, and concentrations that often exceed realistic dietary intake. These variations complicate direct comparison across studies and limit the generalisability of individual findings to human health. Therefore, interpretation of the evidence requires careful consideration of study design, physiological relevance of exposure levels, and the specific endpoints assessed.

- **Carrageenan**

CGN is a sulphated polysaccharide widely used in the food industry for its gelling, thickening and stabilising properties. It is an emulsifier derived from red seaweed, commonly used in beverages to stabilise emulsions [31]. Due to its extensive use, increasing evidence has raised concerns regarding its impact on intestinal health, particularly through interactions with the gut microbiota and the induction of inflammatory responses [29]. Importantly, these effects appear to be largely dose-dependent, reflecting exposure levels rather than the compound’s intrinsic toxicity [30].

CGN exposure has also been associated with disruption of the mucus barrier, a critical physical and biochemical interface between luminal microbes and the epithelium. Reduction in mucus thickness may increase microbial proximity to epithelial cells, facilitating enhanced microbial–host interactions and activation of immune signalling pathways. This mucus impairment, combined with epithelial permeability changes, may predispose the host to low-grade inflammation, particularly under conditions of repeated or chronic exposure. However, these findings are often derived from studies employing high concentrations or exposure periods that may not accurately reflect typical human dietary intake, limiting direct translational relevance [15].

The promotion of pro-inflammatory signalling pathways, notably through the activation of NF-κB and increased production of inflammatory mediators such as IL-8, is due to interactions in gut microbial composition and metabolism, rather than a direct interaction with epithelial cells. At the molecular level, CGN-induced inflammation has been linked to activation of innate immune signalling pathways within intestinal epithelial cells [29]. Experimental studies indicate that CGN can activate Toll-like receptor-mediated signalling, leading to downstream activation of the NF-κB pathway and subsequent upregulation of pro-inflammatory cytokines, including IL-8. This signalling cascade promotes epithelial stress responses, increased permeability, and recruitment of immune cells to the intestinal mucosa. In parallel, CGN-associated alterations in gut microbiota composition may further amplify inflammatory signalling through increased exposure of epithelial cells to microbial-derived pro-inflammatory metabolites and pathogen-associated molecular patterns, thereby reinforcing a feed-forward inflammatory loop [29].

Research on CGN primarily focuses on its two main types: iota-(i-)CGN and kappa-(k-)CGN, which differ in their gelling properties. While i-CGN forms soft, heat-reversible gels, k-CGN forms strong, heat-stable gels. Due to safety concerns, multiple studies have investigated the potential adverse effects of CGN on gut health (Table 2). In vivo studies duration periods vary significantly, ranging from 90 days [32,33] to lifelong administration, and concentrations ranging from 0.1% to 25% (*w*/*v*). Studies using concentrations between 0.1% and 5.0% have reported ulcerative lesions, diarrhoea, and bowel damage [34,35,36]. While in vitro research is limited, the existing studies display a wide range of concentrations and testing periods. The longest trials in cell models lasted 96 h [37], with CGN administration at 1.0 and 0.1% (*w*/*v*), with most studies reporting CGN-induced inflammation [38]. Animal studies provide the most robust evidence regarding the adverse intestinal effects of CGN exposure. Based on the data summarized in Table 2, reported outcomes of CGN consumption are heterogeneous and depend on dose, CGN type, species, and exposure duration.

Severe adverse outcomes, including hepatic alterations and mortality, have primarily been reported in studies using extremely high dietary concentrations (>15–25%), which are considered non-physiological and unlikely to represent realistic human exposure scenarios [34,39]. At moderate levels (<5%), the majority of subchronic and chronic studies in rodents, pigs, and non-human primates reported no significant effects on growth, behaviour, organ weights, or intestinal morphology. However, mild gastrointestinal effects, such as soft stools or transient diarrhoea, were occasionally observed [32,33,40].

Studies using mg/kg body weight doses, closer to estimated human intake levels, generally showed no compound-related toxicity. However, some evidence suggests that λ-CGN may alter gut microbiota composition and metabolic activity without overt clinical diseases [29,34,39]. Overall, the animal studies evidence indicates that potential adverse effects of CGN are dose-dependent and species-specific, with limited toxicological relevance at exposure levels.

Human evidence on CGN indicates limited but relevant findings. Clinical data suggest that consumption of food-grade CGN may aggravate disease activity and shorten relapse time in patients with ulcerative colitis in remission, indicating a potential risk for susceptible populations [41,42,43]. A controlled dietary intervention study reported that exclusion of CGN from the diet was associated with reduced disease activity and inflammatory markers in patients with ulcerative colitis, indicating a potential role of CGN in promoting intestinal inflammation in susceptible individuals [44,45].

In contrast, in vitro studies using human intestinal cell lines generally show no cytotoxicity, oxidative stress, or intestinal permeability, although CGN has been shown to activate pro-inflammatory signalling pathways (TLR-4) [46]. Overall, human data does not demonstrate overt toxicity in healthy models but suggests a possible pro-inflammatory effect under specific pathological conditions, highlighting the need for further controlled studies in humans.

Across various experimental conditions, most studies indicated that CGN fosters an inflammatory environment [33,37,41,47]. However, some studies have found no significant effects [32,33,48]. As summarised in Table 2, these results underscore the need for personalised guidance on CGN intake and further investigation into its gastrointestinal health and effects.

Overall, while animal and in vitro studies provide mechanistic insight into potential inflammatory effects of CGN, the wide variability in study design, exposure levels, and endpoints underscores the need for caution when extrapolating these findings to human health risk assessment. Human evidence remains limited and suggests that potential adverse effects may be restricted to susceptible populations rather than the general healthy population.

**ii.**  **Carboxymethyl Cellulose**

CMC is a water-soluble polysaccharide derived from cellulose, widely used as a food emulsifier for improving texture and stability [46]. It cannot be directly digested or degraded by enzymes of the GIT; therefore, its biological effects are thought to arise predominantly from indirect interactions with the gut microbiota rather than from host enzymatic metabolism [46]. In recent years, increasing attention has been given to its potential effects on gastrointestinal health, particularly on intestinal inflammation and alterations in the gut microbiota [46]. Experimental evidence suggests that certain members of the gut microbiota, especially taxa within the phylum Bacteroidota, may contribute to the metabolism of synthetic cellulose derivatives such as CMC under specific conditions, potentially facilitated by the presence of fermentable dietary fibers that stimulate microbial enzymatic activity [46].

Mechanistically, CMC appears to exert its effects predominantly through microbiota-dependent pathways rather than direct epithelial toxicity. Experimental models suggest that CMC can alter the spatial organisation of gut microbiota, promoting closer microbial encroachment toward the epithelial surface and disrupting the protective mucus layer. This altered microbial localisation may impair mucus barrier function and increase epithelial exposure to microbial products, thereby facilitating low-grade immune activation [49]. Additionally, CMC-induced shifts in microbial metabolic activity, including altered SCFA profiles, may compromise epithelial energy supply and barrier maintenance, indirectly promoting inflammatory susceptibility [44,50].

Notably, the interpretation of CMC-associated effects is complicated by limited study numbers, small sample sizes, and marked heterogeneity in experimental conditions, including differences in microbiota composition, dietary background, and exposure duration, all of which influence observed outcomes.

Reported studies on CMC concentrations range from 0.1% to 10.0% [51,52]. Most in vivo studies in mice indicate that dietary exposure to 1.0% CMC induces chronic low-grade inflammation and colitis, accompanied by alterations in microbiota localisation, composition, and pro-inflammatory potential [46,53,54].

These findings suggest that CMC may disrupt host–microbiota interactions, contributing to intestinal inflammatory responses; however, not all studies have reported adverse outcomes; safety trials using higher concentrations (2.5–10.0%) did not show statistically significant treatment-related effects on evaluated physiological parameters, highlighting inconsistencies across experimental models and endpoints [51,55,56,56].

Findings from in vitro and ex vivo models further support a potential microbiota-mediated mechanism. Studies using human intestinal cell lines (HT29-MTX and HEpG2) reported a strong pro-inflammatory profile following CMC exposure [44,50]. At the same time, microbiota-focused systems demonstrated persistent alterations in microbiota composition and increased pro-inflammatory potential at concentrations as low as 0.1% [44,57].

Taken together, current evidence suggests microbiota-mediated effects of CMC in experimental models; however, inconsistencies across studies and the scarcity of human data highlight significant uncertainty regarding long-term health implications at realistic dietary exposure levels (Table 3).

**Table 2:** Studies on the effects of CGN consumption.

CGN Type	Model	Period	Concentration	Effects	Footnote	Reference
κ/λ-CGN from *C. cripus*	Mouse	70 days	0.0, 5.0, 10 or 20% (*w*/*v*)	Mice with the highest dose died.		[42]
		23–143 days	2.0, 5.0, 10, 15, or 20% (*w*/*v*)	No effects on appearance or behaviour were observed in male and female Osborne–Mendel or Sprague–Dawley rats fed 5% (*w*/*v*).		[58]
	Pigs	83 days	0.0, 50.0, 200.0, or 500 mg/kg bw per day	No compound-related deaths were seen, and the behaviour, appearance, and feed intake of the animals remained normal.	In one pig receiving 200 mg/kg bw per day and two pigs receiving 500 mg/kg bw per day, areas of epithelial infolding were observed, along with infiltration of the colonic lamina propria by macrophages and lymphocytes. However, these findings were considered reversible.	[47]
	Rats	Lifelong administration	0.0, 0.1, 5.0, 15.0, or 25.0% (*w*/*v*)	Evidence of hepatic cirrhosis, only at the 25% concentration, with no effect on mortality.		[40]
			0.5, 2.5, or 5.0% (*w*/*v*)	Soft stool consistency at the beginning of the experiment.		[48]
		183 days	4.0% (*w*/*v*)	There was no effect on growth rate, and the caecum and colon were normal on gross and microscopic examination.	The rat caecum is significantly larger than the human caecum, providing a greater surface area for bacterial activity and therefore increasing the potential for absorption in rodents because of bacterial degradation, leading to observable results.	[47]
κ-CGN	Rats	28–90 days	1.0 or 5.0% (*w*/*v*)	No changes were observed in the stools of rats receiving 1% of either carrageenan. At 5% concentration, rats had loose stools.		[59]
	Humans	90 days	100 mg	Carrageenan consumption may aggravate ulcerative colitis disease activity and reduce the interval to relapse in patients who are in clinical remission.		[34]
ι -CGN from *E. spinosum*	Guineapigs	20 days	1.0% (*w*/*v*)	Two of four treated animals had ulcerative lesions in the caecum. The control group remained healthy.		[32]
		56 days	5.0% (*w*/*v*)	Formation of multiple pinpoint caecal and colonic ulcerations.		[32]
	Rhesus monkeys	49–77 days	1.0 and 5.0% (*w*/*v*)	There were effects of gastrointestinal disturbances at 5%.		[40]
	Infant baboons	112 days	0.0, 1.0, or 5.0% (*w*/*v*)	No effect was seen on organ or body weights, characteristics of the urine and faeces.	After death, it was possible to observe intestinal flood loss caused by λ-CGN.	[29]
	Rats	112 days	5.0% (*w*/*v*)	Formation of multiple pinpoint caecal and colonic ulcerations.		[59]
		56 days	5.0% (*w*/*v*)	Slight diarrhea.		[32]
λ-CGN	Rats	365 days	3.400–3.900 mg/kg (bw) per day	No observation of adverse effects.	The study focuses on reproduction effects, demonstrating that there was no difference related to the dosage, but within each generation, the fertility decreased with consumption of CGN.	[30]
	Guinea pigs	--	2.0% (*w*/*v*)	Bowel lesions first (from 2 to 6 weeks). Colonic lesions developed after 8 weeks.		[24]
	Mice	56 days	1.70, 8.30 or 41.7 mg/kg	λ-CGN may create an environment that favours inflammation by altering gut microbiota composition and gut bacterial metabolism.		[38]
	A Caco-2 absorption model	--	100, 500 and 1000 mg/mL	No cytotoxicity or CGN permeability was observed.	This cell line is tumour-derived and, therefore, may not be representative of in vivo intestinal epithelium.	[42]
	Two cell lines (HEK293)	1906 days	0.1, 1.0, and 10.0 mg/mL	No effect on oxidative stress was observed after 24 h.	The cell line used in this study differs from the human colon epithelial (NCM460) cells.	[41]
	Human intestinal cells	3 days	1.0 μg/mL	Inflammation and colitis. Carrageenan triggers TLR-4, which mediates intestinal inflammation.		[43]

**Table 3:** Studies on the effects of CMC consumption.

Test Type	Model	Period	Concentration	Effects	Footnote	Reference
**Animal** **(in vivo)**	Mice	77 days	1.0% (*w*/*w*)	Increased disease incidence, leading to chronic inflammation and colitis.		[55]
		84 days	1.0% (*w*/*v*)	Alteration of the microbiota localisation, composition, and pro-inflammatory potential.		[53]
		91 days	1.0% (*w*/*v*)	It confirms the induction of low-grade inflammation.		[46]
		--	2.5, 5.0 and 10.0% (*w*/*v*)	No statistically significant or treatment-related adverse effects on any of the parameters evaluated in the safety trials.	The CMC used in the studies was produced from maise husk agrowaste to meet global pharmaceutical standards.	[54]
	Zebrafish embryos	--	5000 ppm for microinjection application.	It can lead to important effects on lipid metabolism by causing changes in the expression of some genes associated with obesity.		[51]
**Cell Line** **(in vitro)**	MiniBioReactor Array model	--	0.1% (*w*/*v*)	Induced a lasting, seemingly detrimental impact on microbiota composition and function.		[52]
	HT29-MTX and Hep G2 cells	--	1.56 and 25.0 mg/mL	Presented a strong pro-inflammatory profile.		[57]
	(M-SHIME) model	--	1.00, 0.50, 0.25 or 0.10% *w*/*v*	Acted directly upon the human microbiota to increase its pro-inflammatory potential.		[57]

**iii.**  **Comparative Perspective and Critical Assessment**

The continuous consumption of food additives such as CGN and CMC, particularly amid rising consumption of processed foods, has been linked to multiple gut health issues. Both exhibit distinct but overlapping mechanisms of interaction with gut microbiota and host intestinal physiology. CGN shows a more pronounced association with direct inflammatory responses, whereas CMC predominantly affects microbial ecology and intestinal barrier function. Nevertheless, these effects create an environment that promotes inflammation, disrupts lipid metabolism and alters gastrointestinal health.

Emerging evidence suggests that the impact of CGN and CMC on the gut microbiota may be context-dependent, with more pronounced effects observed under specific clinical conditions. In individuals with IBD, dietary exposure to CGN has been associated with exacerbation of disease activity and shortened remission periods, potentially through enhanced activation of pro-inflammatory signalling pathways and increased microbial-derived immune stimulation. Clinical and ex vivo studies indicate that, in these populations, CGN may amplify existing epithelial barrier dysfunction and immune dysregulation, thereby promoting a pro-inflammatory gut environment [24,29,32,33,34,38,40,40,47,48,58,59].

Similarly, CMC exposure appears to be more relevant in conditions characterised by altered mucus barrier integrity and microbial instability, such as metabolic syndrome and obesity-associated low-grade inflammation. Experimental models suggest that CMC-induced changes in microbial spatial organisation and reduced mucus barrier protection may be particularly detrimental in hosts with pre-existing metabolic or inflammatory dysfunction, where microbial resilience and barrier repair mechanisms are already compromised [46,51,52,53,54,55,57].

In contrast, findings from studies in healthy models generally indicate limited or subclinical effects, highlighting that the adverse microbiota-related consequences of CGN and CMC may disproportionately affect vulnerable populations rather than the general healthy population. Collectively, these observations underscore the importance of considering host health status when evaluating the gut microbiota-modulating effects of food emulsifiers [41,42,43,57].

The major limitation across studies is the lack of harmonised experimental designs, particularly regarding dosage, exposure, duration, and microbial assessment methods. Additionally, most available data derive from simplified models that may not fully capture the complexity of human dietary patterns and gut ecosystems.

A key aspect of gut modulation and potential dysbiosis associated with CGN and CMC exposure is the alteration of microbial populations, particularly shifts within the Bacteroidota and Firmicutes phyla. Reported changes included increased abundance of genera such as *Bacteroides*, *Faecalibacterium*, *Akkermansia*, *Enterococcus*, *Fusobacterium*, *Parvimonas*, *Gemella*, *Leptotrichia*, and *Roseburia*, as well as representatives of the genera *Enterobacter* (within the Enterobacteriaceae family) and *Bacillus* (from the Bacillales order). Conversely, decreases have been noted in genera such as *Clostridium*, *Bifidobacterium*, *Faecalibacterium*, *Blautia*, and members of the genus *Lachnospira* (from the Lachnospiraceae family) [44,50], which are clustered with intestinal homeostasis.

Specific bacterial species implicated in CGN- and CMC-associated dysbiosis, which may favour inflammatory processes, are summarised in Table 4. These taxa warrant prioritised attention in future mechanistic and human intervention studies, as they represent key microbial markers linking food additive exposure to inflammation-derived gut dysfunction.

These species require prioritised attention when studying the effects of additives on the gut microbiota, as they can create an environment that supports inflammation and are directly associated with the consumption of these additives. Overall, the converging evidence highlights the need for standardised, microbiota-centred risk assessment frameworks and underscores the importance of evaluating food additives not only in isolation but also within the context of realistic dietary patterns and long-term consumption.

## 6. Hands-On Exploration of CGN and CMC Effects In Vitro

As an initial screening approach, in vitro gastrointestinal simulation models are widely used to investigate the effects of CGN and CMC on the structure and function of the gut microbiota. These models provide controlled, reproducible conditions that enable mechanistic exploration of additive–microbiota interactions while reducing time, cost, and ethical constraints associated with in vivo experimentation. Importantly, in vitro systems allow isolation of microbiota-driven effects, thereby facilitating mechanistic insights into how CGN and CMC may modulate microbial metabolism, ecological stability, and host-relevant metabolic outputs.

Within this context, in vitro fermentation models address key knowledge gaps that cannot be readily explored with in vivo studies, including the direct microbiota-mediated effects of CGN and CMC, the impact of repeated or prolonged exposure under controlled conditions, and the identification of early functional markers of dysbiosis prior to overt pathology [60]. By enabling precise control over substrate concentration, exposure duration, and microbial inoculum, these models allow systematic evaluation of dose–response relationships and temporal microbial adaptation, which are difficult to disentangle in animal or human studies. Validation of findings is typically achieved through comparison with established microbial and metabolic signatures reported with in vivo models and, where available, human intervention studies. Nevertheless, these systems do not fully replicate host immune responses or epithelial–microbiota crosstalk and should therefore be interpreted as complementary tools rather than standalone predictors of human health outcomes [60,61].

In vitro fermentation models are particularly suited to investigate microbial fermentability, metabolic fluxes, and functional changes induced by CGN and CMC. As these additives are not directly hydrolysed by human digestive enzymes, their biological activity is hypothesized to arise primarily through microbiota-dependent mechanisms [46]. These include selective microbial utilisation, shifts in community composition, altered SCFA production, and changes in metabolites linked to intestinal inflammation and epithelial integrity [60].

Typically, this approach involves a combination of simulated upper gastrointestinal digestion followed by anaerobic colonic fermentation, allowing dynamic profiling of bacterial populations and their metabolic outputs under controlled conditions [60].

- **Fermentation Assay**

Fermentability assays provide critical mechanistic insights into whether CGN and CMC can be metabolised by gut microorganisms and how such metabolism influences microbial ecology and functionality. Key parameters include bacterial proliferation, organic acid production, pH variation, and ammonia generation. These readouts reflect microbial energy harvesting, proteolytic versus saccharolytic activity, and ecosystem stability—factors tightly linked to intestinal homeostasis and inflammatory risk [60].

Alterations in SCFA profiles, particularly reductions in butyrate or increases in proteolytic metabolites such as ammonia, may indicate a shift toward dysbiosis and compromised epithelial support. Although both in vivo and in vitro studies have examined the effects of CGN and CMC, significant knowledge gaps remain regarding the microbial and metabolic consequences of prolonged exposure, which in vitro systems are well positioned to address [60].

Fermentation experiments may be conducted using batch (fed-batch) or continuous systems. Continuous models enable long-term, steady-state operation, whereas fed-batch systems allow precise modulation of substrate availability while reducing metabolite accumulation. Fed-batch systems are widely applied in gut microbiota research, as periodic substrate renewal enables temporal assessment of microbial adaptation, resilience, and metabolic reprogramming in response to repeated additive exposure [60,61,62].

**ii.**  **Sample Selection**

Human faecal samples are commonly used as microbial inocula due to their accessibility, non-invasiveness, and microbial richness. While faecal samples do not fully capture mucosa-associated communities, they are considered representative of luminal microbiota and provide a practical model for assessing microbiota-driven effects of CGN and CMC. Given that approximately 55–60% of stool mass consists of bacteria, faecal inocula offer a robust platform for evaluating additive-induced shifts in microbial composition and metabolic activity under standardized conditions [46,54].

**iii.**  **Additive Supplementation**

Previous studies have typically employed fermentation periods of approximately 48 h. To address limitations of acute exposure models, extended fermentation strategies that incorporate medium renewal at defined time points may better simulate repeated dietary intake. Such approaches enable assessment of microbial adaptation, cumulative metabolic effects, and longer-term dysbiotic trends induced by CGN and CMC [60,62].

**iv.**  **Metabolite Analysis**

Metabolite profiling is central to the mechanistic interpretation of in vitro fermentation outcomes. The gut microbiota metabolises non-digestible carbohydrates and proteins into SCFAs (acetate, propionate, and butyrate), as well as intermediate and proteolytic metabolites such as succinate, lactate, branched-chain fatty acids, and ammonia. These metabolites directly influence epithelial integrity, immune signalling, and inflammatory tone [60].

Monitoring SCFA production, pH shifts, gas release, and ammonia concentrations provides functional insight into whether CGN and CMC promote metabolic patterns associated with homeostasis or pathological states, such as excessive proteolysis, epithelial stress, or pro-inflammatory signalling [31,62].

**v.**  **Human GUT Simulation**

Advanced in vitro gastrointestinal models replicate key physicochemical and physiological conditions of the human GIT. These models integrate the study of gut digestion and absorption processes with the assessment of nutrient bioavailability and bioaccessibility [62]. The process can be broken down into three sequential steps: (1) simulating gastrointestinal digestion, (2) mimicking intestinal absorption, and (3) performing colonic fermentation studies. The application of INFOGEST, with further in vitro gastrointestinal models, can provide data on bacterial quantification, organic acid production, and NH_4_^+^ measurement [60].

Application of such dynamic systems allows simultaneous evaluation of bacterial composition, metabolite production, and ammonia levels, making them particularly relevant for studying chronic exposure scenarios and additive–microbiota interactions. Nevertheless, despite their increased physiological relevance, these models remain simplifications of the human gut and should be interpreted in conjunction with in vivo evidence [60].

## 7. Conclusions and Future Perspectives

This review highlights the growing body of evidence indicating that widely used food additives, such as CGN and CMC, can influence gut microbiota composition, intestinal barrier integrity, and inflammatory responses. Although these additives are generally regarded as safe based on traditional toxicological endpoints, emerging data suggest that their chronic consumption, particularly within diets rich in ultra-processed foods, may have unintended consequences for gut health.

CGN and CMC exhibit distinct yet converging mechanisms of action. CGN has been more consistently associated with direct pro-inflammatory effects, including activation of innate immune signalling pathways and epithelial stress responses. In contrast, CMC primarily affects microbial spatial organisation and the integrity of the mucus barrier, thereby indirectly promoting dysbiosis and low-grade inflammation. Despite these mechanistic differences, both additives contribute to an intestinal environment that favours microbial imbalance, barrier dysfunction, and inflammatory susceptibility. The mechanism underlying its inflammatory potential remains under investigation, but current evidence suggests that CMC alters gut microbial diversity and induces colitis-like symptoms in experimental models. Despite growing concerns regarding the health implications of CGN and CMC, gaps remain in our understanding of their long-term effects, particularly in human populations. Further studies are needed to clarify safe consumption thresholds and to assess potential cumulative effects from prolonged exposure.

A critical limitation in the current literature is the predominance of in vitro and animal studies, alongside significant heterogeneity in experimental design, including differences in additive type, molecular characteristics, dosage, and exposure duration. Human data remains scarce and highly variable, underscoring the need for well-designed, long-term intervention studies that account for individual variability in the gut microbiota composition and its susceptibility.

The observed shifts in microbiota composition suggest that consumption of CGN and CMC may contribute to gut dysbiosis by fostering an inflammatory environment. The dominance of Bacteroidota and Firmicutes, along with the reduction in beneficial bacteria such as *Bifidobacterium* and *Faecalibacterium*, is typical in conditions associated with gut microbiota imbalance. These changes are particularly concerning, given the role of these bacterial communities in maintaining intestinal homeostasis, modulating immune responses, and producing SCFAs that support gut health. Understanding these mechanisms is crucial for assessing the long-term implications of emulsifier consumption on human health.

From a food safety and regulatory perspective, these findings highlight the limitations of current safety assessment frameworks, which primarily rely on acute toxicity and genotoxicity endpoints and do not routinely consider gut microbiota modulation or chronic low-dose exposure. Incorporating microbiome-related outcomes, realistic dietary exposure scenarios, and long-term effects into regulatory risk assessment could improve the evaluation of emulsifier safety.

From a public health standpoint, the widespread and cumulative exposure to CGN and CMC through ultra-processed foods raises concerns, particularly for vulnerable populations such as individuals with IBS/IBD, metabolic disorders, or altered gut microbiota. These findings support the need for evidence-based dietary guidance that considers not only nutrient composition but also additive exposure within modern food systems.

In conclusion, while CGN and CMC remain valuable additives, their widespread and long-term consumption warrants greater scientific scrutiny. Future research should prioritise harmonised methodologies, mechanistic clarity, and human-relevant models to better define safe-use conditions and support evidence-based dietary guidance in the context of modern food systems.

## Figures and Tables

**Table 1:** Food additives intended for re-evaluation after 2020.

Food Additive Type	Name	EFSA Number
**Antioxidant**	Tartaric Acid	E334
	Sodium Tartrate	E335
	Potassium Tartrate	E336
	Sodic Potassium Tartrate	E337
	Calcium Tartrate	E354
	Metataric Acid	E353
**Colorant**	Calcium Carbonate	E170
	Vegetal Carbon	E153
	Esters of Acetic Acid	E472a, E472b, E472d, E472e, E472f
**Texturisers**	Stearyl Tartrate	E483
	Carrageenan	E407
	Carboxymethyl Cellulose	E466

**Table 4:** Dysbiosis by CGN and CMC consumption that can favour inflammation, adapted from [44,50].

Increase		Decrease
*Escherichia coli*	*Enterotoxigenic Bacteroides fragilis*	*F. prausnitzii*
*Desulfovibrio*	*Clostridium hatheway*	*Blautia faecis*
*Clostridium cocleatum*	*Clostridium symbiosum*	*Roseburia inulinivorans*
*Clostridium thermosuccinogenes*	*Bacteroides vulgatus*	*Clostridium lavalense*
*Coprobacillus catenaformis*	*Veillonella* *denticariosi*	*Clostridium coccoides*
*Ruminococcus torques*		*Clostridium leptum*
*Ruminococcus bromii-like bacteria*		*Akkermansia muciniphila*
*Helicobactor pylori*		
*Streptococcus bovis*		
*Fusobacterium nucletum*		

## Data Availability

No new data were created or analyzed in this study. Data sharing is not applicable to this article.

## Abbreviations

The following abbreviations are used in this manuscript:

SCFA	Short Chain Fatty Acid
IBD	Inflammatory Bowel Disease.
IBS	Irritable Bowel Syndrome
GIT	Gastrointestinal Tract
AEIC	Adherent-Invasive *Escherichia coli*
CRC	Colorectal Cancer
ETBT	Enterotoxigenic Bacteroides *fragilis*
BFT	Bacteroides *fragilis* Toxin
T2DM	Type 2 Diabetes Mellitus
CGN	Carrageenan
CMC	Carboxymethyl Cellulose
EFSA	European Food Safety Authority

## References

[1] Adak A., Khan M.R. (2018). An insight into gut microbiota and its functionalities. Cell. Mol. Life Sci..

[2] Magne F., Gotteland M., Gauthier L., Zazueta A., Pesoa S., Navarrete P., Balamurugan R. (2020). The firmicutes/bacteroidetes ratio: A relevant marker of gut dysbiosis in obese patients?. Nutrients.

[3] Meeting and World Health Organization Meeting and World Health Organization 2007. https://cdn.who.int/media/docs/default-source/biologicals/cell-substrates/cells.final.mtgrep.ik.26_sep_07.pdf?sfvrsn=3db7d37a_3.

[4] Gomez-Arango L.F., Barrett H.L., Wilkinson S.A., Callaway L.K., McIntyre H.D., Morrison M., Nitert M.D. (2018). Low dietary fiber intake increases *Collinsella* abundance in the gut microbiota of overweight and obese pregnant women. Gut Microbes.

[5] Abiega-Franyutti P., Freyre-Fonseca V. (2021). Chronic consumption of food-additives lead to changes via microbiota gut-brain axis. Toxicology.

[6] Zhou X., Qiao K., Wu H., Zhang Y. (2023). The Impact of Food Additives on the Abundance and Composition of Gut Microbiota. Molecules.

[7] Mariat D., Firmesse O., Levenez F., Guimarăes V., Sokol H., Doré J., Corthier G., Furet J.-P. (2009). The firmicutes/bacteroidetes ratio of the human microbiota changes with age. BMC Microbiol..

[8] Zafar H., Saier M.H. (2021). Gut *Bacteroides* species in health and disease. Gut Microbes.

[9] Wexler A.G., Goodman A.L. (2017). An insider’s perspective: Bacteroides as a window into the microbiome. Nat. Microbiol..

[10] Stojanov S., Berlec A., Štrukelj B. (2020). The Influence of Probiotics on the Firmicutes/Bacteroidetes Ratio in the Treatment of Obesity and Inflammatory Bowel disease. Microorganisms.

[11] Cao Y., Liu H., Qin N., Ren X., Zhu B., Xia X. (2020). Impact of food additives on the composition and function of gut microbiota: A review. Trends Food Sci. Technol..

[12] Bäckhed F., Ding H., Wang T., Hooper Lv Young Koh G., Nagy A., Semenkovich C.F., Gordon J.I. (2004). The Gut microbiota as an Environmental Factor That Regulates Fat Storage. Proc. Natl. Acad. Sci. USA.

[13] Curtis H., Blaser M.J., Dirk G., Kota K.C., Rob K., Liu B., Wang L., Sahar A., White J.R., Badger J.H. (2012). Structure, function and diversity of the healthy human microbiome. Nature.

[14] Belkaid Y., Hand T.W. (2014). Role of the microbiota in immunity and inflammation. Cell.

[15] Rinninella E., Raoul P., Cintoni M., Franceschi F., Miggiano G.A.D., Gasbarrini A., Mele M.C. (2019). What is the healthy gut microbiota composition? A changing ecosystem across age, environment, diet, and diseases. Microorganisms.

[16] Frank D.N., St Amand A.L., Feldman R.A., Boedeker E.C., Harpaz N., Pace N.R. (2007). Molecular-phylogenetic characterization of microbial community imbalances in human inflammatory bowel diseases. Proc. Natl. Acad. Sci. USA.

[17] Center M.M., Jemal A., Ward E. (2009). International Trends in Colorectal Cancer Incidence Rates. Cancer Epidemiol. Biomark. Prev..

[18] Quaglio A.E.V., Grillo T.G., De Oliveira E.C.S., Di Stasi L.C., Sassaki L.Y. (2022). Gut microbiota, inflammatory bowel disease and colorectal cancer. World J. Gastroenterol..

[19] Krogius-Kurikka L., Lyra A., Malinen E., Aarnikunnas J., Tuimala J., Paulin L., Mäkivuokko H., Kajander K., Palva A. (2009). Microbial community analysis reveals high level phylogenetic alterations in the overall gastrointestinal microbiota of diarrhoea-predominant irritable bowel syndrome sufferers. BMC Gastroenterol..

[20] Karsa L., Lignini T., Patnick J., Lambert R., Sauvaget C. (2010). The dimensions of the CRC problem. Best Prac. Res. Clin. Gastroenterol..

[21] Srivastava N., Ibrahim S., Chattopadhyay J., Arbab M. (2023). Frontmatter. The Gut Microbiota in Health and Disease.

[22] Gao R., Gao Z., Huang L., Qin H. (2017). Gut microbiota and colorectal cancer. Eur. J. Clin. Microbiol. Infect. Dis..

[23] Hou H., Chen D., Zhang K., Zhang W., Liu T., Wang S., Dai X., Wang B., Zhong W., Cao H. (2022). Gut Microbiota-Derived Short-Chain Fatty Acids and Colorectal Cancer: Ready for Clinical Translation?. Cancer Lett..

[24] Wu W., Zhou J., Xuan R., Chen J., Han H., Liu J., Niu T., Chen H., Wang F. (2022). Dietary κ-carrageenan facilitates gut microbiota-mediated intestinal inflammation. Carbohydr. Polym..

[25] Gurung M., Li Z., You H., Rodrigues R., Jump D.B., Morgun A., Shulzhenko N. (2020). Role of gut microbiota in type 2 diabetes pathophysiology. EBioMedicine.

[26] Zhou Z., Sun B., Yu D., Zhu C. (2022). Gut Microbiota: An Important Player in Type 2 Diabetes Mellitus. Front. Cell. Infect. Microbiol..

[27] Castaner O., Goday A., Park Y.-M., Lee S.-H., Magkos F., Shiow S.-A.T.E., Schröder H. (2018). The Gut Microbiome Profile in Obesity: A Systematic Review. Int. J. Endocrinol..

[28] Carocho M., Barreiro M.F., Morales P., Ferreira I.C. (2014). Adding Molecules to Food, Pros and Cons: A Review on Synthetic and Natural Food Additives. Compr. Rev. Food Sci. Food Saf..

[29] Necas J., Bartosikova L. (2013). Carrageenan: A review. Vet. Med..

[30] Collins T., Black T., Prew J. (1977). Long-term effects of calcium carrageenan in rats—I. Effects on reproduction. Food Cosmet. Toxicol..

[31] Jabeen F., Ahmad R., Mir S., Awwad N.S., Ibrahium H.A. (2025). Carrageenan: Structure, properties and applications with special emphasis on food science. RSC Adv..

[32] Grasso P., Sharratt M., Carpanini F.M.B., Gangolli S.D. (1973). Studies on Carrageenan and Large-bowel Ulceration in Mammals.

[33] Watt J., Marcus R. (1971). Carrageenan-induced ulceration of the large intestine in the guinea pig. Gut.

[34] McKim J.M., Wilga P.C., Pregenzer J.F., Blakemore W.R. (2015). The common food additive carrageenan is not a ligand for Toll-like- Receptor 4 (TLR4) in an HEK293-TLR4 reporter cell-line model. Food Chem. Toxicol..

[35] Benitz K.F., Golberg L., Coulston F. (1973). Intestinal Effects of Carrageenans in the Rhesus Monkey.

[36] Mcgill H.C., Mcmahan C.A., Wigodsky H.S., Sprinz H. (1977). Carrageenan in Formula and Infant Baboon Development. Gastroenterology.

[37] Poulsen E. (1973). Short-term peroral toxicity of undegraded carrageenan in pigs. Food Cosmet. Toxicol..

[38] Bhattacharyya S., Shumard T., Xie H., Dodda A., Varady K.A., Feferman L., Halline A.G., Goldstein J.L., Hanauer S.B., Tobacman J.K. (2017). A Randomized Trial of the Effects of the No-Carrageenan Diet on Ulcerative Colitis Disease Activity. Nutr. Healthy Aging.

[39] Grasso P., Gangolli S.D., Butterworth K.R., Wright M.G. (1975). Studies on Degraded Carrageenan in Rats and Guinea-Pigs.

[40] Abraham R., Benitz K.-F., Mankes R., Rosenblum I. (1985). Chronic and Subchronic Effects of Various Forms of Carrageenan in Rats. Ecotoxicol. Environ. Saf..

[41] McKim J.M. (2014). Food Additive Carrageenan: Part I: A Critical Review of Carrageenan in Vitro Studies, Potential Pitfalls, and Implications for Human Health and Safety. Crit. Rev. Toxicol..

[42] Borthakur A., Bhattacharyya S., Anbazhagan A.N., Kumar A., Dudeja P.K., Tobacman J.K. (2012). Prolongation of carrageenan-induced inflammation in human colonic epithelial cells by activation of an NFκB-BCL10 loop. Biochim. Biophys. Acta (BBA)-Mol. Basis Dis..

[43] Costa E.M., Silva S., Pereira C.F., Ribeiro A.B., Casanova F., Freixo R., Pintado M., Ramos Ó.L. (2023). Carboxymethyl Cellulose as a Food Emulsifier: Are Its Days Numbered?. Polymers.

[44] Komisarska P., Pinyosinwat A., Saleem M., Szczuko M. (2024). Carrageenan as a Potential Factor of Inflammatory Bowel Diseases. Nutrients.

[45] Borsani B., De Santis R., Perico V., Penagini F., Pendezza E., Dilillo D., Bosetti A., Zuccotti G.V., D’auria E. (2021). The Role of Carrageenan in Inflammatory Bowel Diseases and Allergic Reactions: Where Do We Stand?. Nutrients.

[46] Mondal I.H., Yeasmin M.S. (2016). Toxicity Study of Food-Grade Carboxymethyl Cellulose Synthesised from Maize Husk in Swiss Albino Mice. Int. J. Biol. Macromol..

[47] Rhee M., Pittz E., Abraham R. (1981). Effect of Combinations of Irideae Carrageenan and Cellulose on the Absorption of Some Nutrients from the Alimentary Tract of Rats. Ecotoxicol. Environ. Saf..

[48] Nilson H.W., Wagner J.A. (1959). Feeding test with carrageenin. Food Res..

[49] Chassaing B., Compher C., Bonhomme B., Liu Q., Tian Y., Walters W., Nessel L., Delaroque C., Hao F., Gershuni V. (2021). Randomized Controlled-Feeding Study of Dietary Emulsifier Carboxymethylcellulose Reveals Detrimental Impacts on the Gut Microbiota and Metabolome. Gastroenterology.

[50] de Carvalho N.M., Oliveira D.L., Saleh M.A.D., Pintado M., Madureira A.R. (2021). Preservation of Human Gut Microbiota Inoculums for In Vitro Fermentations Studies. Fermentation.

[51] Naimi S., Viennois E., Gewirtz A.T., Chassaing B. (2021). Direct Impact of Commonly Used Dietary Emulsifiers on Human Gut Microbiota. Microbiome.

[52] Chassaing B., Van de Wiele T., De Bodt J., Marzorati M., Gewirtz A.T. (2017). Dietary Emulsifiers Directly Alter Human Microbiota Composition and Gene Expression Ex Vivo Potentiating Intestinal Inflammation. Gut.

[53] Viennois E., Bretin A., Dubé P.E., Maue A.C., Dauriat C.J., Barnich N., Gewirtz A.T., Chassaing B. (2020). Dietary Emulsifiers Directly Impact Adherent-Invasive *E. coli* Gene Expression to Drive Chronic Intestinal Inflammation. Cell Rep..

[54] Baran A., Sulukan E., Türkoğlu M., Ghosigharehagaji A., Yildirim S., Kankaynar M., Bolat I., Kaya M., Topal A., Ceyhun S.B. (2020). Is Sodium Carboxymethyl Cellulose (CMC) Really Completely Innocent? It May Be Triggering Obesity. Int. J. Biol. Macromol..

[55] Chassaing B., Koren O., Goodrich J.K., Poole A.C., Srinivasan S., Ley R.E., Gewirtz A.T. (2015). Dietary Emulsifiers Impact the Mouse Gut Microbiota Promoting Colitis and Metabolic Syndrome. Nature.

[56] Zangara M.T., Ponti A.K., Miller N.D., Engelhart M.J., Ahern P.P., Sangwan N., McDonald C. (2022). Maltodextrin Consumption Impairs the Intestinal Mucus Barrier and Accelerates Colitis Through Direct Actions on the Epithelium. Front. Immunol..

[57] Cohen S.M., Ito N. (2002). A Critical Review of the Toxicological Effects of Carrageenan and Processed Eucheuma Seaweed on the Gastrointestinal Tract. Crit. Rev. Toxicol..

[58] Tomarelli R.M., Tucker W.D., Bauman L.M., Savini S., Weaber J.R. (1974). Nutritional quality of processed milk containing carrageenan. J. Agric. Food Chem..

[59] Martino J.V., Van Limbergen J., Cahill L.E. (2017). The Role of Carrageenan and Carboxymethylcellulose in the Development of Intestinal Inflammation. Front. Pediatr..

[60] Trilokesh C., Uppuluri K.B. (2021). Biobutanol from Lignocellulosic Biomass and Microalgae: Scope, Technology, and Economics. Sustainable Biofuels.

[61] de Carvalho N.M., Oliveira D.L., Costa C.M., Pintado M., Madureira A.R. (2022). Can Supplemented Skim Milk (SKM) Boost Your Gut Health?. Fermentation.

[62] Louis P., Flint H.J. (2017). Formation of Propionate and Butyrate by the Human Colonic Microbiota. Environ. Microbiol..

